# Neonatal death audits at Kgapane Hospital, Limpopo province

**DOI:** 10.4102/safp.v65i1.5815

**Published:** 2023-12-22

**Authors:** Gert J.O. Marincowitz, Clara Marincowitz

**Affiliations:** 1 Department of Family Medicine, Faculty of Health Sciences, University of Limpopo, Mankweng, South Africa; 2 Department of Psychiatry, Faculty of Health Sciences, University of Stellenbosch, Tygerberg, South Africa; 3 SA Medical Research Council, Cape Town, South Africa

**Keywords:** neonatal death, perinatal, audit, rural health, district hospital

## Abstract

**Background:**

Neonatal deaths (NNDs) are a global public health challenge, predominantly affecting low- and middle-income countries. The causes of most NNDs are preventable. Therefore, this study reviewed perinatal clinical audit data at Kgapane Hospital over a 4-year period with a special focus on the factors associated with NNDs.

**Methods:**

File audits were performed for all NNDs occurring at Kgapane Hospital and its catchment area from 2018 to 2021. The data from these audits were analysed to identify factors associated with NNDs.

**Results:**

The NND rate for the study period was 12.6/1000 live births. In this study (*n* = 236), 90% of the deaths could be associated with four factors, namely prematurity (44%); intrapartum complications (19%) including asphyxia, meconium aspiration and breech deliveries; neonatal infections (16%) of which human immunodeficiency virus (HIV) positivity was the most prevalent; and foetal congenital abnormalities (11%). The modifiable factors included inadequate intrapartum foetal monitoring; delays in management interventions; instances where no attempts were made to refer patients for specialised care, or where no beds were available at the next level of care; patient-related factors; and inadequate adherence to management protocols, such as for the management of prematurity and HIV.

**Conclusion:**

Understanding factors associated with NNDs can guide health worker training and improvement strategies to reduce this heart-breaking complication of pregnancy.

**Contribution:**

Family physicians working in rural hospitals are also responsible for newborn care. Understanding the factors associated with NNDs will guide them to focus training and develop improvement strategies to reduce these preventable deaths.

## Introduction

Neonatal deaths (NNDs) are a global public health challenge predominantly affecting low- and middle-income countries.^1^ In high-income countries, the early neonatal mortality rate is about 3/1000 live births for infants weighing 500 g or more.^2^ Comparatively, in sub-Saharan Africa, the early neonatal mortality rate is about 27/1000 live births for infants weighing 500 g or more.^1^

The first month of life is the most vulnerable period for child survival, with 2.4 million newborns dying in 2020.^1^ That year, nearly half (47%) of all under-five deaths occurred in the newborn period, an increase from 1990 (40%). This is because of the global level of under-five mortality declining faster than that of neonatal mortality.^1,3,4^

Sub-Saharan Africa has the world’s highest neonatal mortality rate, accounting for 43% of global newborn deaths. It is followed by central and southern Asia (23/1000 live births), with 36% of global newborn deaths.^1^ Internationally, preterm birth, intrapartum-related complications (birth asphyxia), congenital abnormalities and infections are the leading causes of NNDs.^5,6,7,8,9,10,11,12,13^ According to the Saving Babies report (2014–2016), the common identifiable primary causes of NND in South Africa are spontaneous preterm labour (48.1%), intrapartum hypoxia (24.2%), neonatal infections (11.2%), and foetal abnormalities (9.1%). Unknown causes accounted for 1.8% and the remaining NNDs were caused by various other conditions including trauma.^3,4^

Considering that most of the causes of neonatal mortality reported in the literature are preventable, it is of interest to describe the audit findings from Kgapane Hospital highlighting preventable causes of neonatal mortality, which could aid in developing improvement strategies. Therefore, the aim of this study was to analyse findings from perinatal death audits conducted at Kgapane Hospital between 2018 and 2021 to identify factors associated with NNDs, and describe the women who lost neonates.

## Research methods and design

Kgapane Hospital is a medium sized district hospital with 178 beds, in the Mopani District of South Africa’s Limpopo province. It serves a population of 230 000 people and has 21 clinics in its catchment area. The hospital has a maternity section delivering around 5000 babies annually with 250 or more deliveries performed at the 21 clinics. Monthly, there are 16 full-time midwives allocated to the maternity section, including labour ward, ante- and post-natal wards resulting in a midwife to birth ratio of 3.5.^14^

Individual file audits were performed on all perinatal deaths that occurred at Kgapane Hospital and its catchment area between February 2018 and October 2021. Data from these audits were captured for each individual perinatal death and used to do a retrospective observational study investigating the causes of NND. All deaths of neonates delivered at Kgapane Hospital and its catchment area were included in the study. Deaths were excluded where the records were not available for audit.

The data extraction tool developed to extract information from the audit documents was based on the Perinatal Problem Identification Programme (PPIP) data collection sheet, which is a standardised and tested tool in South Africa.^4^ All maternal demographic, obstetric and health data, pregnancy outcomes and foetal factors, plus the factors associated with death were extracted from the audit reports and captured by the researcher on a password-protected Excel® spreadsheet. Subsequently, the entered data were checked and cleaned and analysed descriptively.

### Ethical considerations

Ethical clearance for the research was obtained from the Research and Ethics Committee of the University of Limpopo (TREC/61/2023: IR), and permission was acquired from the Limpopo Provincial Department of Health (LP 2023-03-012) as well as Kgapane Hospital management. A waiver of consent was received from the CEO of Kgapane Hospital as secondary data were used for the study with no implications for the patients. For confidentiality, only patient codes were used while capturing the data.

## Results

A total of 254 NNDs occurred from 01 February 2018 to 31 October 2021 at Kgapane Hospital and its surrounding clinics. Women who delivered in transit to the hospital or at home and came to the hospital thereafter were also included. The total number of births for this period was 20 562, which resulted in an early neonatal mortality rate of 12.6/1000 live births.

Of the 254 recorded NNDs in the period, audits were performed on 236 of the records with 18 missing. Data from all the available NND records were included in the study analysis.

**FIGURE 1:**
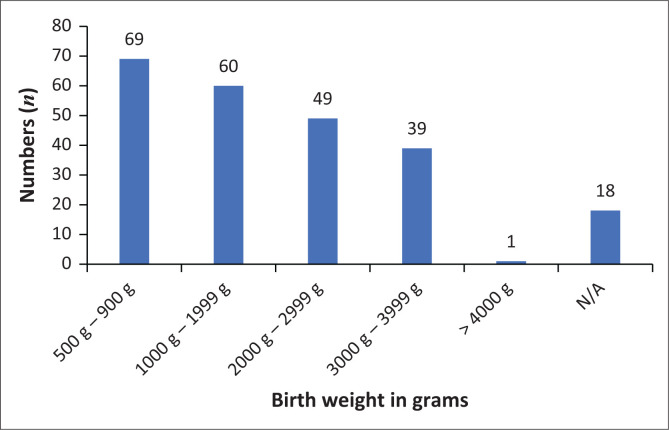
Birth weights of neonates who died (*N* = 236).

The demographic information of women who had NNDs is presented in detail in Table 1. The mean age was 27.25 years; 36% never had a living child before (including those who only had previous miscarriages or stillbirths) and for 32% it was their first pregnancy. Of these women, 45 had experienced one or more previous miscarriages. The majority (86%) delivered in the hospital. Thirty-eight (16%) of the newborns who died were from multiple pregnancies. The distribution of the birth weights of neonates who died is presented in Figure 1.

**TABLE 1a:** Demographic data of women who had neonatal deaths (*N* = 236).

Age	*n*	%	Mean	Median
< 20 years	34	14.4	27, 25	27
20–30 years	71	30.1	-	-
30–40 years	59	25.0	-	-
> 40 years	13	5.5	-	-
Unknown	59	25.0	-	-

BANC, basic antenatal care at primary care level; HRC, high-risk clinic at the hospital.

**TABLE 1b:** Demographic data of women who had neonatal deaths (*N* = 236).

Variable	Gravidity		Parity	
	*n*	%	*n*	%
0	NA	NA	88	37.3
1	76	32.2	59	25.0
2	56	23.7	37	15.7
3	36	15.3	27	11.4
4	27	11.4	12	5.1
5	22	9.3	2	0.8
> 5	12	5.0	4	1.7
Unknown	7	3.0	7	3.0

BANC, basic antenatal care at primary care level; HRC, high-risk clinic at the hospital; NA, not applicable.

**TABLE 1c:** Demographic data of women who had neonatal deaths (*N* = 236).

Variable	Previous miscarriage		Previous Stillbirth		Previous NND		Previous infant death		Multiple pregnancy	
	*n*	%	*n*	%	*n*	%	*n*	%	*n*	%
Yes	43	18.2	4	1.7	3	1.3	11	4.7	38	16.1
No	190	80.5	229	97.0	230	97.5	222	94.1	195	82.6
Unknown	3	1.3	3	1.3	3	1.3	3	1.3	3	1.3

BANC, basic antenatal care at primary care level; HRC, high-risk clinic at the hospital.

**TABLE 1d:** Demographic data of women who had neonatal deaths (*N* = 236).

ANC attended at least once	*n*	%
BANC	206	87.3
Never	23	9.7
HRC	55	23.3
Unknown	7	3.0

ANC, antenatal care; BANC, basic antenatal care at primary care level; HRC, high-risk clinic at the hospital.

**TABLE 1e:** Demographic data of women who had neonatal deaths (*N* = 236).

Place of delivery	*n*	%
Hospital	202	85.6
Clinic	13	5.5
Home	16	6.8
In transit	5	2.1

BANC, basic antenatal care at primary care level; HRC, high-risk clinic at the hospital.

Of the women who experienced neonatal loss, 87.3% attended basic antenatal care at least once, and 23.3% of them also attended the high-risk clinic at the hospital. Of the remainder, only 9.7% never attended any form of antenatal care and for 3% no data were available.

**FIGURE 2:**
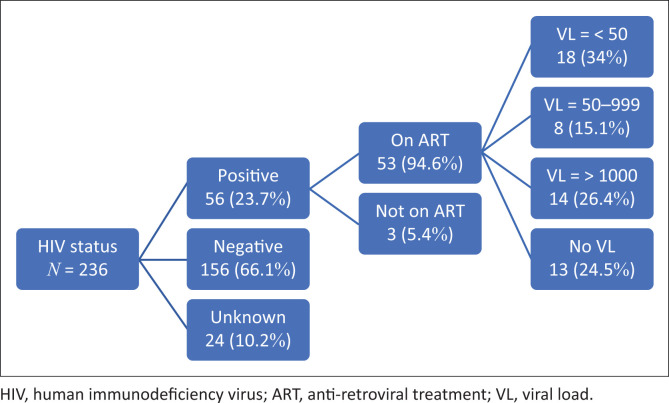
Human immunodeficiency virus data for women with neonatal deaths.

The human immunodeficiency virus (HIV)-status of 56 of the women was positive (in 24, the HIV status was unknown), and 53 of them were on treatment. Of those with known viral loads, 18 had a viral load of less than 50 copies/mL, in 8 the viral load was between 50 and 999, and 14 had viral loads of 1000 and above (Figure 2).

**FIGURE 3:**
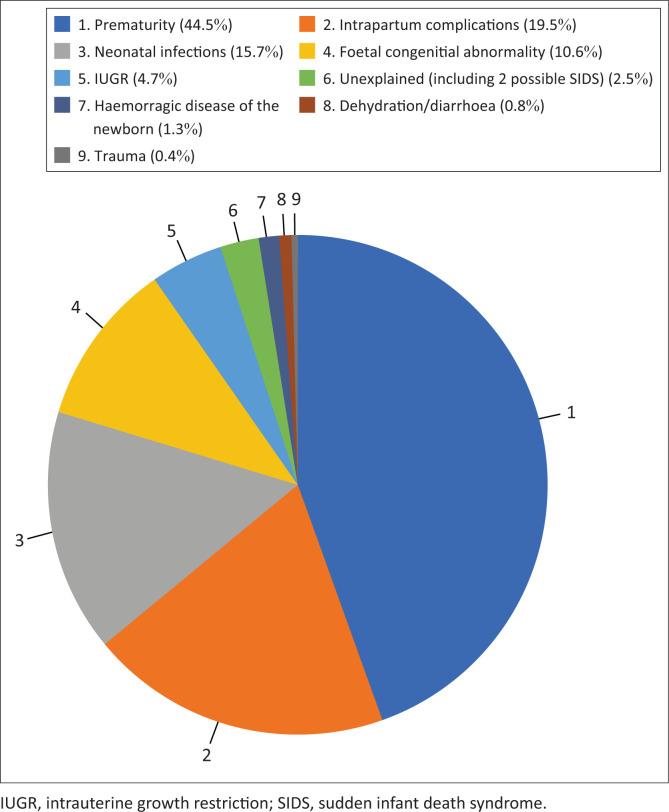
Factors associated with neonatal deaths.

Ninety per cent of the NNDs could be ascribed to the following four conditions: prematurity, intrapartum complications, neonatal infections, and foetal congenital abnormalities (Table 2 and Figure 3).

**TABLE 2:** Factors associated with neonatal deaths (*N* = 236).

Factors	Sub-totals (*n*)	Group totals (*n*)	Percentage (%)
Prematurity	-	105	44.5
Intrapartum complications	-	46	19.5
Birth asphyxia	36	-	-
Meconium aspiration syndrome	6	-	-
Breech	4	-	-
Infections	-	37	15.7
Neonatal sepsis	19	-	-
HIV with VL unsuppressed (10 premature)	16	-	-
COVID-19	1	-	-
Congenital syphilis	1	-	-
Foetal congenital abnormality	-	25	10.6
Intrauterine growth restriction (IUGR)	-	11	4.7
Unexplained (including two possible SIDS)	-	6	2.5
Haemorrhagic disease of the newborn	-	3	1.3
Dehydration/diarrhoea	-	2	0.8
Trauma	-	1	0.4

HIV, human immunodeficiency virus; VL, viral load; COVID-19, coronavirus disease 2019; SIDS, sudden infant death syndrome.

## Discussion

In this study, 90% of the deaths were associated with four causes, namely, prematurity (44%); intrapartum complications (19%) including asphyxia, meconium aspiration and breech deliveries; neonatal infections (16%) of which HIV positivity was the most prevalent; and foetal congenital abnormalities (11%). It is concerning that only 18 (34%) of the women on antiretroviral treatment had suppressed viral loads of below 50. This is far below the national target of a 95% viral load suppression rate.^15^

The four major contributing factors causing NND which were identified in this study have been echoed by multiple other studies.^6,7,8,9,10,11,12,13^ Most of these studies used data based on the South African National PPIP data, reported by public health facilities of whom most were also rural. They identified very similar proportions for these four causes.^3,4,5,7,12,13^ Of note is that a few studies, conducted in Nepal, Pakistan and India, identified considerably higher rates of infections.^8,9,11^ However, this could be due to differences in interpretation of the cause of death. It is therefore possible that the lower rate of neonatal infections reported in the current study may be due to some NNDs being caused by unnoticed infections, because premature labour is frequently precipitated by infections.^1,11^

According to Rhoda et al.^12^ the most important management strategies to reduce NNDs are administering antenatal corticosteroids for preterm labour, labour and delivery management, prevention of mother-to-child transmission of HIV, the use of oral rehydrating solution for babies with diarrhoea, handwashing with soap, and case management of severe neonatal infection. While these strategies are protocol at Kgapane Hospital, the results of this study suggest that they should be encouraged and monitored to a greater extent.

The inadequate intrapartum monitoring is frequently due to insufficient staffing and resources, including equipment such as cardiotocographs.^3,4,5,12,13,14,16^ The recommended midwife to birth ratio in low resource settings by the International Federation of Gynecology and Obstetrics (FIGO) is 1.71 births per midwife, with an ideal FIGO ratio of 15.2.^14^ In contrast, at the maternity section where this study was conducted, the midwife to birth ratio was 3.5.

## Conclusion

The importance of perinatal audits lies in the fact that it is widely accepted that knowing the causes of neonatal mortality helps to identify preventable factors for improvement.^16^ This study found four leading factors associated with NNDs at a rural, level-one hospital, namely prematurity, intrapartum asphyxia, neonatal infections, and foetal congenital abnormalities. These can largely be attributed to inadequate management of premature labour, poor intrapartum monitoring, delays in management interventions and referral to specialised care. Inadequate staffing and limited resources are likely a contributing factor.

### Recommendations

At Kgapane Hospital and its catchment area, care can be improved with more training and implementation of key guidelines^17,18^; better intrapartum monitoring; timely referrals of patients who require specialised care; and responding promptly to signs and symptoms which might indicate risk factors, especially premature labour. The most important topics for training are the prevention and management of prematurity, the management of neonatal sepsis and the management of HIV-positive women to ensure that they are virologically suppressed. Additionally, attention should be directed at improving staffing and resource allocation. Adequate resources and well-trained personnel are essential to improve effective intrapartum monitoring and the care of premature neonates.^19^ A continuous audit process can help to identify deficiencies and monitor improvement;^16^ these tools are readily available.^17^

### Limitations

Limitations include the fact that retrospective data were used and that recordkeeping was frequently not at the expected standard. Furthermore, because the file audits were conducted by the researcher who also worked in the ward at that time, the results could be compromised with bias introduced into the results. Finally, even though searched for by the records clerk on several occasions, 18 patient files remained missing and were not audited.
